# Glycoside Hydrolases and Glycosyltransferases from Hyperthermophilic Archaea: Insights on Their Characteristics and Applications in Biotechnology

**DOI:** 10.3390/biom11111557

**Published:** 2021-10-21

**Authors:** Khadija Amin, Sylvain Tranchimand, Thierry Benvegnu, Ziad Abdel-Razzak, Hala Chamieh

**Affiliations:** 1 Laboratory of Applied Biotechnology, Azm Center for Research in Biotechnology and Its Applications, Lebanese University, Mitein Street, Tripoli P.O. Box 210, Lebanon; khadija.a.amin@hotmail.com (K.A.); ziad.abdelrazzak@ul.edu.lb (Z.A.-R.); 2 Univ Rennes, Ecole Nationale Supérieure de Chimie de Rennes, CNRS, ISCR-UMR 6226, F-35000 Rennes, France; sylvain.tranchimand@ensc-rennes.fr (S.T.); thierry.benvegnu@ensc-rennes.fr (T.B.); 3 Faculty of Sciences, Lebanese University, Rafic Hariri Campus, Beirut P.O. Box 6573, Lebanon

**Keywords:** CAZymes, glycoside hydrolases, glycosyltransferases, hyperthermophilic archaea, biotechnology

## Abstract

Hyperthermophilic Archaea colonizing unnatural habitats of extremes conditions such as volcanoes and deep-sea hydrothermal vents represent an unmeasurable bioresource for enzymes used in various industrial applications. Their enzymes show distinct structural and functional properties and are resistant to extreme conditions of temperature and pressure where their mesophilic homologs fail. In this review, we will outline carbohydrate-active enzymes (CAZymes) from hyperthermophilic Archaea with specific focus on the two largest families, glycoside hydrolases (GHs) and glycosyltransferases (GTs). We will present the latest advances on these enzymes particularly in the light of novel accumulating data from genomics and metagenomics sequencing technologies. We will discuss the contribution of these enzymes from hyperthermophilic Archaea to industrial applications and put the emphasis on newly identifed enzymes. We will highlight their common biochemical and distinct features. Finally, we will overview the areas that remain to be explored to identify novel promising hyperthermozymes.

## 1. Introduction

Archaea are prokaryotic microorganisms with distinct biochemical and physiological traits and have important ecological significance due to the ability of many archaeal members to thrive in extreme habitats. Due to some of their unusual characteristics, Archaea have largely contributed to the development of novel biotechnological processes to produce highly valuable bioproducts [1]. In particular, hyperthermophilic Archaea that colonize extreme environments of temperature have recently received great interest and constitute promising bioresource of biocatalysts for high-temperature industrial processes [2].

The discovery of CAZymes (carbohydrate-active enzymes) from hyperthermophilic Archaea represents excellent examples of proteins well adapted for biotransformation in conditions where conventional enzymes denature rapidly because of their remarkable thermostability. In addition, many hyperthermophilic proteins show extreme resistance to detergents and some with high tolerance to extreme pH conditions particularly for proteins from hyperthermophilic acidophilic or alkaliphilic Archaea. This makes them ideal for industrial processes requiring harsh conditions of temperature, pH and denaturing conditions [3,4]. CAZymes are active enzymes that display a key role in the biosynthesis, modification, binding and catabolism of carbohydrates and are found in all living organisms [5]. The availability of sequenced genomes allowed the classification of all CAZymes and to cover the data on their genetic, structural, mechanistic, and functional information in a CAZy database that constitutes a worldwide knowledge-based resource about CAZymes [5,6]. In addition, the database provides online access for the classification of CAZymes into different families based on the similarity of their amino acid sequences, as follows: the carbohydrate esterases, the polysaccharide lyases, enzymes with auxiliary activities, the carbohydrate binding modules, the glycoside hydrolases and the glycosyltransferases. The two latter constitute the largest classes and their biochemical and enzymatic properties have been extensively studied [6,7].

In this review, we will emphasize mainly on GHs and GTs from hyperthermophilic Archaea. These enzymes, distinguished by their ability to synthesize and break the glycosidic bond, display interesting properties that allowed them to be studied and exploited as new tools in biotechnology for many industrial applications [8]. This is reflected by their high optimal thermostability which provides numerous biotechnological benefits over their mesophilic or psychrophilic enzymes: (i) they are more easily purified by heat treatment when expressed in a heterologous host. Many archaeal hyperthermophilic proteins were characterized by their production in recombinant systems due to the accessibility of their genes from available sequenced genomes and the ease with which they can be recombinantly produced and purified from heterologous hosts such as *Escherichia coli*. (ii) they are more resistant to chemical denaturants, (iii) they display higher rigidity that is required for high protein thermostability, and they can be used at a higher temperature which can decrease viscosity, (iv) provide faster reaction rates [9,10,11]. This enhanced thermostability is accomplished by the presence of a higher number of hydrophobic amino acids in the enzyme’s core and an increased number of ionic interactions, surface charges and salt bridges.

## 2. Hyperthermophilic GHs from Archaea

### 2.1. General Features and Reaction Mechanisms

**Figure 1:**
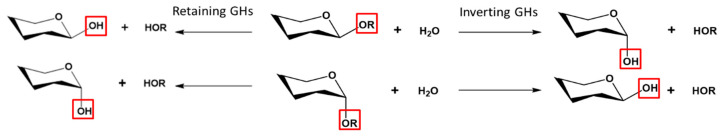
Mechanistic classification of GHs showing Inverting and Retaining GHs [20].

Glycoside hydrolases, referred to as glycosidases, and glycosyl hydrolases are abundant in almost all living organisms [12,13,14] and are involved in metabolism, antibacterial defense, and pathogenesis [15,16]. They are primarily involved in the hydrolysis and/or transglycosylation of glycosidic bonds present in glycosides, glycans and glycoconjugates [5]. By referring to the CAZy database and based on similarities in amino acid sequences, GHs are grouped into 171 families [17] (last access July 2021). Some families share conserved catalytic mechanisms and structure and are classified into clans, named by letters from A to N, according to their three-dimensional folding [18]. GHs are also classified based on the either retaining or inverting mechanism. Indeed, GHs require two catalytic amino acid residues, usually glutamic or aspartic acids, which are usually located 6–11 Å apart and act as a general acid/proton donor or as a base/nucleophile, respectively, to catalyze the hydrolysis of glycosidic bonds [12,19]. Based on the spatial arrangement of these residues, the hydrolysis can take place through either retention or inversion of the anomeric carbon configuration (Figure 1) [20].

### 2.2. Glycoside Hydrolases (GHs) in the Genomes of Hyperthermophilic Archaea

**Figure 2:**
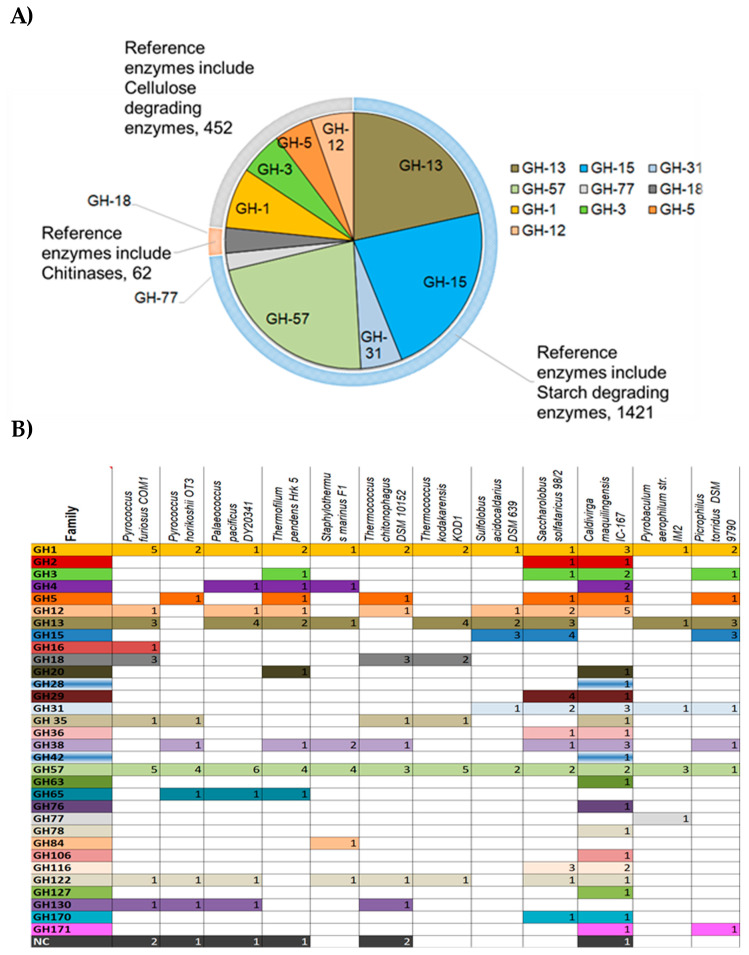
Classification of most abundant archaeal GHs [20]. (**A**) Diagram showing abundancy of GHs in Archaea and their repartition into different enzyme classes. Sequences from archaeal genomes corresponding to each GH family were retrieved from the CAZY database and the number of occurring sequences for each GH family was determined and represented on the pie chart; (**B**) Occurrence of GHs families in representative hyperthermophilic Archaea. NC: Non-classified.

Many sequence-based analyses tried to address the biodiversity of volcanic sites and hot springs with the goal of discovering and characterizing novel hyperthermophilic CAZymes to explore their industrial and biotechnological potential [21,22,23]. GHs are abundant in archaeal genomes as summarized in Figure 2. From the 420 analyzed archaeal genomes, 2800 protein sequences corresponding to GH families were found in the CAZy database. Among these protein sequences, the GH-15 family in the inverting GHs is the most widespread in Archaea (Figure 2A) and comprises enzymes such as glucoamylase, glucodextranase, α,α-trehalase, and dextran dextrinase family (615 protein sequences/420 analyzed archaeal genomes). This family is abundant in some archaeal representatives such as halophiles (e.g., *Haloferax* spp.). However, it is not ubiquitous to all hyperthermophiles since it is present in Sulfolobales, Saccharolobales but completely absent from the genomes of the Thermococcales (Figure 2B). Among retaining GHs, GH-13 (417 sequences found/420 analyzed genomes) and GH-57 families (429 sequences found/420 analyzed genomes) predominate in Archaea and are formed of enzymes with various activities acting mainly on starch and pullulan such as α-amylase, amylopullulanases, branching enzymes, pullulanase, cyclomaltodextrin glucanotransferase, cyclomaltodextrinase. These two families are found in all the analyzed hyperthermophilic archaeal genomes and included at least one up to five protein sequence members.

It is interesting to notice that members of the GH1 family are found exclusively in the genomes of hyperthermophilic Archaea (Figure 2B). This family comprises enzymes such as the beta glycosidase, beta glucosidase, beta mannosidase and beta galactosidase.

### 2.3. Biochemical Features and Biotechnological Applications

A wide range of hyperthermophilic archaeal GHs has been investigated and molecularly characterized [24,25,26,27]. They include mainly starch, cellulose and chitin degrading enzymes that have found their way in several industrial applications and exhibit promising opportunities for future applications [28,29]. Beside carbohydrate hydrolysis, GHs can be also involved in glycan synthesis through transglycosylation reactions in which a sugar residue can replace water as the nucleophilic acceptor and be transferred from one glycoside to another, creating novel glycosidic bonds to form oligo- and polysaccharides [30]. Hence, two activities of GHs are exploited in biotechnology: (1) the hydrolysis of glycosidic linkages in glycosides, such as the hydrolysis of long chains of cellulose polymers by endoglucanases into smaller fermentable monosaccharides that can be used as feedstocks to produce biofuels and biorenewable chemicals, and (2) the transglycosylation reactions, which are one of the most promising applications in carbohydrate synthesis [8,31,32]. One example is the transgalactosylation reactions of lactose catalyzed by GH-42 β-galactosidase to produce galactooligosaccharides (GOS) that can be used in various food and pharmaceutical processes [3,33].

#### 2.3.1. Starch Degrading Enzymes

**Figure 3:**
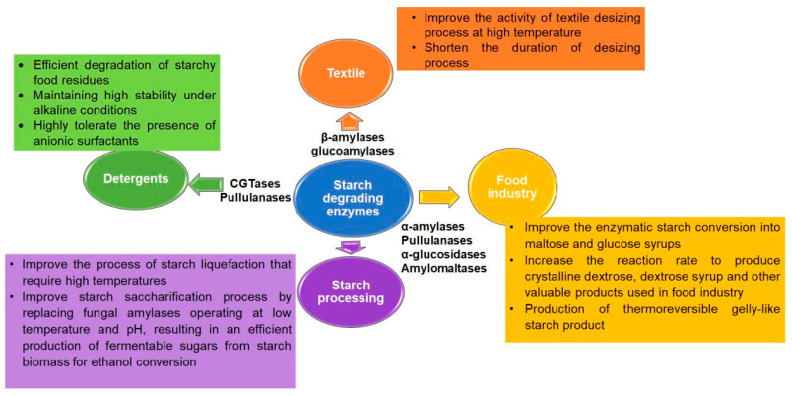
Applications of starch-degrading enzymes. The main applications of hyperthermophilic enzymes from different Archaea in specific industries are summarized in the given text box [8,34,38,39,40,41].

Starch is a complex carbohydrate mostly abundant in plant sources. It is composed of glucose monomers that are linked together via α-1,4 and α-1,6 glycosidic linkages. Starch hydrolysis and modification are exploited in many industrial applications that include food, texture, pulp and paper industries, to produce bioproducts that can be used to make ethanol, amino acids and organic acids as well as fat replacers, aroma stabilizers, texturizing agents and prebiotics [34,35]. Most starch degrading enzymes from Archaea belong to the α-amylase from the GH-13 family, with only a few examples in GH-57. In particular, we shed the light on the amylolytic enzymes from hyperthermophilic Archaea that generally display high thermal stability and are sought to improve the process of starch hydrolysis [34,36]. The use of hyperthermophilic starch degrading enzymes have been also exploited in several industrial domains including food, pharmaceutical and fine chemical industry (Figure 3) [34,37,38].

Some examples of applications of starch degrading enzymes from hyperthermophilic Archaea are discussed below in detail. Multiple forms of starch degrading enzymes have been identified. They mainly consist of α- and β-amylases, isoamylases, glucoamylases and pullulanases and are found to be highly prevalent in thermophiles and hyperthermophiles, implying a key role in primary energy metabolism [34].

##### α-Amylases

Alpha-amylases are widespread in nature, they are mainly produced by microorganisms such as *Bacillus* spp.; *Aspergillus* spp. They act on starch through an endocleavage of the α-1,4 glycosidic bond randomly to produce maltodextrins of lower molecular weight. They have high commercial values and are frequently employed in the early stages of starch processing. Starch processing is very prominent in the food industry and occurs via two steps consisting of liquefaction followed by saccharification in order to produce maltodextrins and glucose syrups. Industrial liquefaction necessitates the highest possible temperature (100–110 °C) to overcome high viscosities and mass transfer problems. During this process, starch (typically from corn for example) is subjected to gelatinization at high temperature and then enzymatically hydrolyzed by α-amylases into dextrin at 95 °C for 1–2 h; As a result, hyperthermophilic enzymes acting at this temperature have proven to be ideal for the liquefaction process. Such enzymes have been discovered in the hyperthermophilic Archaea that belong to *Pyrococcus* sp. ST04 [39], *Pyrococcus woesei* [26,40], *Thermococcus* sp. HJ21 [41,42], *Thermococcus profundus* DT5432 [43,44], *Saccharolobus solfataricus* [45] and the methanogenic Archaea *Methanococcus* *jannaschii* [46]. Most of these enzymes have their optimal activity that resides around 100 °C with a remarkable thermostability. They have the capacity to retain their activity after 4 h of autoclaving (120 °C), allowing them to catalyze hydrolytic reactions and resist harsh operational conditions for many hours [8]. α-amylase from *Pyrococcus furiosus* is currently included in many commercial preparations such as Termamyl/Liquozyme from Novozymes. It has an advantage over the available enzyme from the moderate thermophilic *Bacillus* in that it is calcium independent, allowing it to be used in industrial processes without the addition of calcium [47].

Other amylases participate in maltose production and are involved in the second step of starch saccharification known as malto-saccharification and usually takes place at a temperature at or below 60 °C. These enzymes known as maltogenic amylases (MAase) are versatile and act mainly on the α-1,4 and α-1,6 glycosidic bonds to produce maltoses that are widely used in the confectionery, baking and brewing industries. They belong to the GH-13 family and were reported in the hyperthermophilic Archaeon *Thermoplasma volcanium* GSS1 (*Tp*MA) and Archaeon *Staphylothermus marinus* (*Sm*MA). They exhibit the features of both bacterial MAase and α-amylase and high optimum temperature activity (75 °C for *Tp*MA and 100 °C for *Sm*MA), which make them extremely multifunctional maltogenic amylases to be used in a broad range of applications related to starch and food processing industries [48,49,50,51]. 

##### β-Amylases and Glucoamylases

β-Amylases act on the α-1,4 linkage of α-glucan next to the non-reducing end, resulting in maltose that has β-configuration and exists as the only low molecular weight product, while glucoamylases are used mainly to produce β-glucose from starch.

Both β-amylase and glucoamylase enzymes are required in the starch saccharification process to produce high maltose or glucose syrup and thus are relevantly involved in the food and brewing industries and can be used in the textile industry in starch desizing (Figure 3). Indeed, during the weaving process, fabrics are subjected to high mechanical strength. To prevent the breakage of the yarn during this process, starch must first be applied in a process called sizing but then has to be removed by enzymatic treatment (starch desizing) to subsequently bleach and dye the fabrics. The use of thermophilic amylases that works optimally at 70 °C or higher and pH raging from 5.5 to 6.5 is ideal for the starch desizing process [52].

In hyperthermophilic Archaea, β-amylases and glucoamylases are putative. Only one β-amylase was described from the hyperthermophilic Archaeon *Pyrococcus furiosus* encoded by the ORF PF0870. The corresponding protein belongs to the GH-57 family and is valuable due to its thermostability and optimal activity at 110 °C generating maltose from maltotriose or *p*-nitrophenyl-α-maltopyranoside [53]. An interesting glucoamylase encoded by the *ssg* gene from *Saccharolobus* *solfataricus* was expressed as a recombinant protein in *E. coli*. This tetrameric enzyme is highly thermostable, showing an optimal activity at 90 °C in the pH range from 5.5 to 6.0. The use of this recombinant glucoamylase has proven to be very efficient in the saccharification step because it can operate in the same temperature and pH range of amylases that act during the liquefaction process, making starch processing more economically feasible [38]. Compared to fungal glucoamylases, this thermostable enzyme is also characterized by its substrate preference for maltotriose rather than amylose and amylopectin and constitutes an interesting candidate in the starch processing industry due to its low levels of reversion and transglycosylation activity. Indeed, starch processing at an industrial scale requires high concentrations of solids (>30%) resulting in high glucose concentration affecting the glucoamylase to act toward the formation of the reversion product [38].

Several other glucoamylases have been found in the thermoacidophilic Archaea that belong to *Thermoplasma* *acidophilum*, *Picrophilus oshimae* and *Picrophilus torridus*. Their optimal activity resides at 90 °C but in acidic conditions, at pH 2, and display higher substrate specificity for large molecules [54,55,56]. The thermoactive glucoamylase from *Thermoplasma* *acidophilum* constitutes a promising alternative to fungal glucoamylases for use in the starch industry in the degradation of complex polysaccharides that usually require high temperatures [55].

##### Pullulanases

Pullulanases belong to GH-13 and GH-57 families and are involved in the hydrolysis of the α-1,6 linkages in pullulan, a linear α-glucan polysaccharide formed by *Aureobasidium pullulans* from starch and is made up of maltotriose residues linked together via α-1,6-glycosidic linkages [57]. They can also hydrolyze other branched polysaccharides, such as starch, and can be combined with saccharifying amylases to improve the production of sugar syrup [58]. In particular, thermostable pullulanases have the potential to perform the process of liquefaction-saccharification in only one conversion step without the need of adjusting the pH or temperature to produce various sugar syrups and maltooligosaccharides [9,57,59].

Numerous thermostable pullulanases with optimal temperatures ranging from 90 and 110 °C have been described in the hyperthermophilic archaeal genera *Thermococcus, Pyrococcus, Desulfurococcus*, *Staphylothermus, Pyrodictium* [58,60,61].

Pullulanases are classified into two types based on substrate and reaction product specificity: type I and type II (amylopullulanases).

Type I enzymes have a strong specificity for α-1,6 linkage between glucose residues and are rare in Archaea. An example of type I thermostable pullulanase was isolated from *Pyrodictium abyssi* and is optimally active at 100 °C in an alkaline optimal pH 9 [27]. Due to its increased thermostability in alkaline conditions, this enzyme is an excellent choice for use as an additive in a variety of industrial applications, particularly dishwashing and laundry detergents, because it efficiently removes starches while maintaining the necessary stability under alkaline conditions that most commercial pullulanases cannot [9].

Type II pullulanases, also called amylopullulanases represent the vast majority of archaeal pullulanases and display a dual activity: α-amylase and type I pullulanase, i.e., they are capable of hydrolyzing the α-1,4 or α-1,6 glycosidic linkages of glucose. Among the described thermostable archaeal type II pullulanase, some have particular features. One was described in the hyperthermophilic Archaeon *Thermofilum pendens* (TPApu) and displays an optimal activity between 95–100 °C in acidic optimal pH 3.5. Being the most acidophilic amylopullulanase among thermophilic pullulanases, the high catalytic efficiency of this enzyme towards pullulan and α-cyclodextrin as well as its ability to preferentially debranch the maize amylopectin short side chains, make it an attractive candidate for starch liquefaction and saccharification processes, which are critical for producing maltooligosaccharides with mild sweetness, low osmolality, high water-binding capacity, appropriate viscosity, crystallization inhibition property and bread storage improvement [37,62,63].

Two other thermostable type II pullulanases were described in *Pyrococcus furiosus* and *Thermococcus litoralis* and exhibit high stability between 130 °C and 140 °C [58]. The extreme thermostability of these enzymes and their ability to work in the absence of calcium makes them ideal for use in starch debranching and maltodextrin conversion in maltose or glucose syrups with improved hydrolytic efficiency [9].

##### Isoamylases

Isoamylases cleave the α-1,6 glycosidic linkages to release linear maltooligosaccharides chains such as amylodextrin from both starch and glycogen [58,64,65,66]. Compared to the described thermotolerant α-amylases, few thermostable isoamylases used in the starch industry were studied. However, the use of thermostable isoamylases to perform the debranching process at high temperatures would be beneficial to increase the reaction rate, which in turn reduces the amount of enzyme needed and lowers the cost of biocatalyst [67].

The only archaeal genera with annotated isoamylases in genomic databases belong to the order Sulfolobalus. Two isoamylases belonging to GH-13 family were isolated from the hyperthermophilic Archaea *Sulfolobus tokodaii* and *Saccharolobus* *solfataricus* ATCC 35,092 and heterologously expressed in *E. coli* [64]. Compared to isoamylase from *Saccharolobus* *solfataricus* which is mostly active at 75 °C and pH 5, the isoamylase from *Sulfolobus* *tokodaii* exhibits higher thermostability as it works optimally at 90 °C with a half-life of 200 min in the presence of Mg^2+^, which makes it ideal for simultaneous starch gelatinization and isoamylase hydrolysis for several hours at 90 °C for the production of amylodextrin, resulting in a sugar biobattery with a doubled power output [64]. As a result, treating starch granules with the mentioned thermostable isoamylases is critical for reducing energy consumption and producing linear amylodextrin in a high-viscosity starch slurry [64].

##### α-Glucosidases

α-glucosidases are exo-acting enzymes that are involved in the final stage of starch degradation. They show higher affinity toward small substrates, such as maltotriose, and act on the α-1,4 glycosidic linkages to liberate glucose [9]. In particular, thermostable α-glucosidases have received potential industrial applications for their wide range of substrate specificity and ability to catalyze the transglucosylation reactions. 

Multiple α-glucosidases were isolated from the hyperthermophilic Archaea of the genera *Sulfolobus shibatae*, *Saccharolobus solfataricus*, *Pyrococcus furiosus*, *Pyrococcus woesei*, *Picrophilus torridus* and *Thermococcus litoralis*.

The hyperthermostable intracellular α-glucosidase from *Pyrococcus furiosus* is primarily active between 105 °C and 115 °C in the pH range of 5 to 6 and retains half of its relative activity in pH ranges 4.5 to 7. This enzyme displays a remarkable thermostability: at 98 °C, the half-life is around 48 h [68]. The enzyme from *Saccharolobus* *solfataricus* that belongs to the GH-31 family has comparable operating conditions relative to those of the *Pyrococcus furiosus* enzyme, it is mostly active at 105 °C and pH 4.5, with relative activity of more than 50% at pH 4 to 6, but it is highly resistant to proteolysis and denaturants [69]. Its substrate specificity is also unexpected: while most of the α-glucosidases are inactive on glycogen, the one from *Saccharolobus* *solfataricus* is able to hydrolyze glycogen quite efficiently [70]. The biotechnological potential of thermostable archaeal *α*-glucosidases from both *Saccharolobus* *solfataricus* and *Pyrococcus furiosus* in transglycosylation reactions combined with their thermal activity and stability could be further exploited in biotechnology for the production of food oligosaccharides or for the conjugation of sugars with biologically active materials [71]. The use of these highly thermostable enzymes would provide greater stability and extended enzymatic activity in the operational conditions where higher levels of organics, which are denaturants for conventional enzymes, are typically present [8].

##### Cyclodextrin Glucanotransferases (CGTases)

CGTases are retaining GHs that belong to the GH-13 family and catalyze the cyclization, coupling, disproportionation and hydrolysis reactions leading to cyclic oligoglucosides with α-1,4 glycosidic linkages called cyclodextrins. The resulting starch-derived product displays a wide range of applications that include drug delivery, food, cosmetics, and textiles. Thermostable CGTases are industrially relevant for the production of cyclodextrin. Their thermal stability offers a great advantage in the process of starch liquefaction by eliminating the step of pretreatment with other amylolytic enzymes (such as α-amylase to liquefy the starch) and allows further resistance against detergents and organic solvents [72].

Only a few CGTases were characterized in hyperthermophilic Archaea: one was reported from *Thermococcus kodakaraensis* KOD1(TkCGT) (and from *Thermococcus* sp. strain B-1001) [73], and another from *Pyrococcus furiosus* (PfCGT) [72]. Both are extremely thermostable, with optimal activities at 85 °C and 95 °C for TkCGT and PfCGT respectively, and pH between 5 and 6. Both use starch as substrate, TkCGT is also able to use glycogen but has no activity on pullulan, and the main final product of both enzymes is β-cyclodextrin. It is worth noting that 10 mmol/L of Ca^2+^ slightly increases the optimal temperature of TkCGT from 80 to 85 °C and also increases the stability of the enzyme, its half-life time being 20 min at 100 °C in these conditions. It is worth noting that the TkCGT enzyme is characterized by its hydrolytic activity even when the cyclization activity was abolished by the deletion of the C-terminal domain. This property can confirm the biodiversity of archaeal enzymes, allowing them to be used in a variety of industrial applications [8].

##### Amylomaltases

Amylomaltases catalyze the transfer of an α-1,4-d-glucan segment to another α-1,4-glucan molecule or to glucose. The thermostability of these enzymes would be a desired feature for their biotechnological uses as it increases the half-life of the protein, facilitates its transport and conservation, and allows further resistance against detergents and organic solvents. Different amylomaltases were described in the hyperthermophilic Archaea *Saccharolobus solfataricus* [74], *Thermococccus litoralis* [75], and *Pyrobaculum aerophilum* IM2 [76].

The thermostable amylomaltases isolated from the hyperthermophilic Archaea *Thermococcus litoralis* and *Pyrobaculum aerophilum* (*Py*AMase) are industrially relevant. The latter enzyme belongs to the GH-77 family and is extremely thermostable, retaining 70% of its activity after 55 min at 95 °C at pH 6.7. The enzyme displays an extremely low hydrolysis activity which is 6000-fold lower than the disproportionation activity, comparable to other amylomaltase from the GH-77 family. Its preferred substrate is maltotriose, but disproportionation also occurs on larger oligosaccharides and even on native potato starch. The enzymatic treatment of native potato starch leads to thermoreversible gels with gelatin-like characteristics which could be useful in the food industry [76]. The produced gels exhibit varying melting temperatures, allowing for the creation of a variety of gelling products with a wide tolerance range, making them useful for a variety of industrial applications [8].

Another 4-α-glucanotranferase from a *Pyrobaculum calidifontis*, recently described, has the highest specific activity for such kind of disproportionating enzymes and is one of the most thermostable enzymes described to date. It is characterized by its high thermostability and the potential for starch modification to produce a thermoreversible gel that can substitute the usage of gelatin [77,78].

#### 2.3.2. Cellulose Hydrolyzing Enzymes

Cellulases are cellulose hydrolyzing enzymes that are used to convert cellulosic and lignocellulosic biomass into fermentable sugars. The resulting monomeric products can be then converted into biofuels and other valuable bioproducts by microorganisms [79]. Three types of cellulases are needed for cellulose hydrolysis: endoglucanases, β-glucosidases, and cellobiohydrolases (and exoglucanases), which all act on β-1,4-glycosidic linkages.

**Figure 4:**
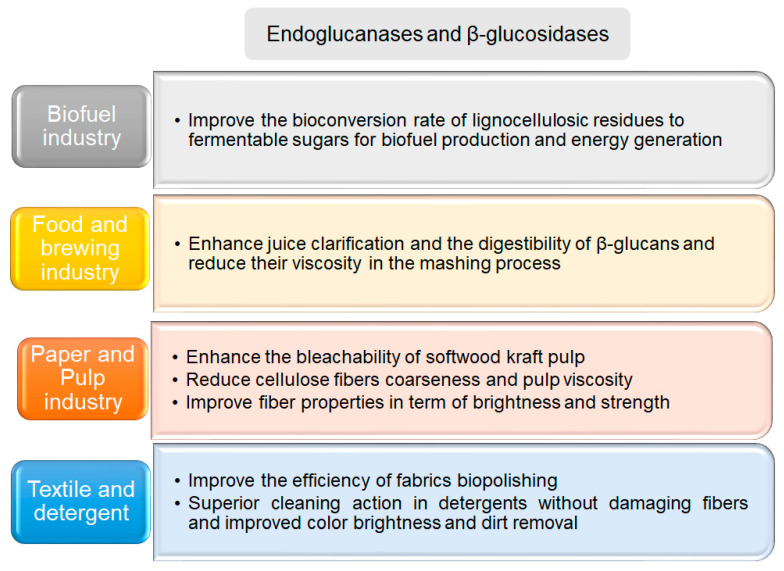
Applications of cellulose degrading enzymes. The main applications of hyperthermophilic enzymes from different Archaea are summarized in the corresponding text box [8,79,81,82,83,84,85].

In particular, the use of extremely thermostable archaeal cellulose hydrolyzing enzymes has shown promise in the production of cellulosic biofuels, the paper and pulp industry, the feed industry, textiles, and detergents (Figure 4). They also increase the yield of biomass conversions from industrial and agricultural waste in the biofuel industry; at high temperatures, cellulose swells, and enzymes working at high temperatures increase the reaction rate, particularly on complex lignocellulosic substrates, as well as the efficiency of their hydrolysis.

Two types of cellulase endoglucanase and β-glucosidase have been isolated from hyperthermophilic Archaea and heterologously expressed in *E. coli* and will be discussed below [80].

##### Endoglucanases

Endoglucanases act on the internal β-1,4 glycosidic bonds of the cellulose chain to produce smaller units [31]. *Pyrococcus horikoshii* was the first hyperthermophilic Archaeon discovered capable of hydrolyzing lignocellulose at temperatures higher than 90 °C. It expresses a multidomain endoglucanase (EGPh, GH-5 family) that belongs to the TIM barrel GH superfamily. This enzyme is most active at 109 °C, with a half-life of 5 h at 100 °C, and highly resistant to denaturation in strong detergents and at high-salt concentrations, making it an excellent candidate for biotechnological applications because it has a higher conversion rate of biomass than its mesophilic counterparts [86].

*P. furiosus* has another endoglucanase, EglA (PF0854), which can hydrolyze cellulose but has much greater activity on short cellobiose oligosaccharides (degree of polymerization [DP] = 5–6), which can be up to 5 × 10^4^ depending on the cellulose properties. Interestingly, this enzyme was able to hydrolyze β-1,4 bonds in mixed-linked-β-d-glucans, with higher efficiency than cellulose, which has a 3 × 10^3^-fold higher specific activity. The enzyme is most active at 100 °C and pH 6, and has a half-life of 40 h at 95 °C [87]. Due to this increased thermostability, the enzyme can be used in the paper processing industry, as well as in the beer brewing industry’s mashing process to reduce the viscosity of barley β-glucan, in the pulp industry to save energy and improve mechanical strength and enhance the bleachability and deinking process of the pulp, and in the feed industry to increase β-glucan digestibility [79,88].

Another endoglucanase was described in *Saccharolobus solfataricus* (SSO1354) and belongs to the GH-12 family. The enzyme performs best at 90 °C in more acidic conditions at pH 4.5, and it can also function in high-salt concentration solutions [89]. This enzyme’s remarkable stability allows it to function in hot solutions that are similar to those used for pretreatment of feedstocks for lignocellulose breakdown [90,91].

##### β-Glucosidases

β-glucosidases catalyze the cleavage of β-glucosidic bonds to release glucose. Because they frequently have broad substrate specificities and can exhibit β–glucosidase, β-galactosidase, β-mannosidase and β-xylosidase activities, some of them are classified as β-glycosidase [92,93]. Thermostable β-glucosidases, in particular, have attracted great industrial importance because of their thermal stability, which allows them to be used in a variety of industrial processes. As they resist denaturation under prolonged hydrolysis conditions, their use in food processing industries and bioconversion of lignocellulosic residues to fermentable sugars for biofuel production and energy generation will be more effective than typical β-glucosidase enzymes. This decreases the amount of enzyme used and increases the yield of the formed product [94,95].

Multiple β–glucosidases have been discovered from hyperthermophilic Archaea. A first example CelB, a GH-1 family enzyme from *Pyrococcus furiosus*, exhibits an optimum activity range of 102 to 105 °C and pH 5, and half-lives of 85 and 13 h at 100 °C and 110 °C, respectively [96]. In addition to β-glucosidase activity, this enzyme shows other β-glucosidase, β-galactosidase, and limited β-mannosidase and β-xylosidase activities. Its heterologous expression allowed for very effective enzyme production, with up to 20% of total *E. coli* cell proteins being produced [97,98]. This hyperthermophilic CelB is a promising candidate for industrial biomass applications for its optimal activity and stability at high temperatures [98]. It displays also high interest in industrial or diagnostic applications and has been used in a variety of processes such as cellulose saccharification, as well as as a transglycosidase to catalyze the synthesis of β-glucosylglycerol or oligosaccharides from lactose [96,98,99,100].

A second GH-1 β-glucosidase, referred as the β-glycosidase PFTG, was identified and characterized from the same Archaeon. Its features are very close to those of the first, with the exception of substrate specificity. This enzyme has also the activities of β-glucosidase, β-galactosidase, β-mannosidase and β-xylosidase, but its β-mannosidase activity is comparable to that of β-glucosidase in this case [101]. The β-glycosidase Asac_1390 from *Acidilobus saccharovorans*, as well as β-glucosidase rSSG from *Sulfolobus shibatae*, are examples of enzymes showing β-glucosidase with very comparable properties [102]. The latter is distinct in that it prefers β-1,3 and β-1,2 over the more common β-1,4 and β-1,6 glycosidic bonds. Its enhanced stability and activity at high temperatures make it industrially important for producing bioethanol from lignocellulosic feedstocks and for glucan degradation in the brewing industry, as it lowers mash viscosity [103,104]. In parallel, using the thermostable β-glycosidase Asac_1390 could also improve the effectiveness of bioconversion processes and make lignocellulosic biomass degradation easier [102].

Finally, the hyperthermophilic Archaeon *Thermofilum pendens* was found to have an enzyme with β-glucosidase activity that belongs to the GH-3 family rather than the GH-1 family. Under extremely acidic conditions, this heat stable enzyme performs best at 90 °C. Its properties are similar to those of the earlier enzymes, with the exception of its optimum pH, which is between 3.5 and 4, while the others have an optimal pH between 5 and 6 [95]. The ability of this enzyme to resist harsh conditions and remain active at high concentrations of dimethyl sulfoxide makes it an attractive source for bioindustrial applications involving bioconversion and transglycosylation reactions to modify the structure of biologically active compounds [95].

#### 2.3.3. Chitinases

Chitins are abundant in the exoskeletons of many groups of invertebrates, in the walls of higher fungi, and in some bacteria. They are unbranched insoluble polymers made up of *N*-acetyl-d-glucosamine repeating units, joined by β-1,4-glycosidic linkages. Chitinases are used on a large industrial-scale to produce pharmaceutical chitooligosaccharides with anti-tumor and wound-healing properties, as well as *N-*acetyl-d-glucosamine, a well-known anti-inflammatory agent used to treat gastrointestinal inflammation disorders and osteoarthritis [105].

**Figure 5:**
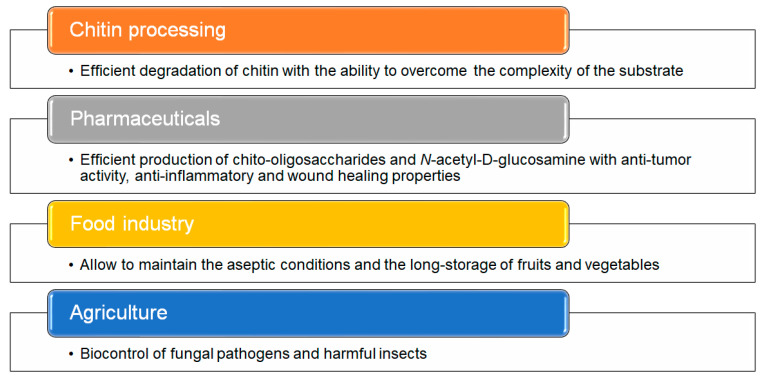
Applications of chitinases. The main applications of hyperthermophilic enzymes from different Archaea are summarized [107,108,109,110].

Thermostable chitin-hydrolyzing enzymes have a significant influence on industrial chitin processing because, when compared to their mesophilic counterparts, they can withstand high temperatures and persist for extended periods in large-scale processes. The high temperature stability of chitinases made them suitable for agricultural use in the biocontrol of fungal pathogens and harmful insects via chitin coat degradation [106]. Furthermore, high thermostability is recommended during food processing to maintain aseptic conditions and control the storage of fruits and vegetables by inhibiting the attack of fungal pests without requiring refrigeration (Figure 5) [9,107,108].

There have been limited studies on archaeal chitinases. Four chitinases from hyperthermophilic Archaea have been characterized: Tk-ChiA from *Thermococcus kodakarensis* KOD1, Tc-ChiD from *Thermococcus chitinophagus*, PF-ChiA from *Pyrococcus furiosus* and Pk-ChiA from *Pyrococcus kodakaraensis* KOD1 [111,112,113].

All these enzymes display common features: they share two active sites from the GH-18 family in a unique polypeptide for the enzyme from *Pyrococcus furiosus*, and two to three chitin-binding sites for the chitinases from *Thermococcus* spp. The two catalytic domains seem to have slightly different catalytic activities; one is chitobiosidase and the other is endo-chitinase. The optimal temperatures for all the enzymes are between 85 and 90 °C, with pH ranging from slightly acidic, between 4 and 5 for Tk-ChiA, Tc-ChiD and Pk-ChiA enzymes, to neutral, between 6.5 and 7 for PF-ChiA chitinase.

The two chitinases from *Thermococcus* spp. are highly thermostable, with half-lives ranging from 48 min for *T. chitinophagus* to 4 h for *T. kodakarensis* at 100 °C. Tk-ChiA is unique in that it has an affinity for both chitin and cellulose, and it can hydrolyze cellulose with 150% of the relative activity on chitosan. The described dual catalytic activity of Tk-ChiA, as well as its remarkable thermostability, provide numerous advantages in industrial applications, making this enzyme a promising hyperthermophilic chitinase for shell treatment and efficient straw waste degradation to reduce environmental risks and pollution [107,110,112].

However, many aspects, such as the low-yield purification process of crude enzyme and production costs, are still limiting the applications of these hyperthermophilic chitinases, and further studies are required to overcome these obstacles and yield economically feasible thermostable chitinases with improved activity and stability.

### 2.4. Metagenomics of Extreme Environments for the Discovery of GHs from Hyperthermophilic Archaea

Hyperthermophilic Archaea have recently received great interest in metagenomic studies of extreme environments and constitute promising bioresource for high-temperature industrial processes. Several studies have been performed to analyze the diversity of microbial communities in hot spring systems using the metagenomic datasets that allow to study the unculturable, but significant microorganisms, to gain new insights for novel archaeal CAZymes [23,114].

A metagenomic study has been performed to investigate the biodiversity of two main mud/water pools in solfatara Pisciarelli, Agnano (Naples, Italy) and has led to the discovery of two novel CAZymes represented by GH5_19 β-mannanase/β-1,3-glucanase and NAD+-dependent GH-109 subfamilies that have been biochemically characterized from a previously unknown Archaeon and have shown a hemicellulose and unreported β-*N*-acetylglucosaminide/β-glucoside specificity, respectively [21,22]. These biocatalysts would represent ideal candidates for biotechnological applications and will display promising applications in next-generation biorefineries in the pretreatment approach of lignocellulosic biomass by increasing the efficiency of the industrial process and improving final yields for more efficient biofuel conversion [21].

Different culture-dependent approaches coupled to metagenomic studies have been also performed to analyze the microbial diversity in extreme hot habitats to discover novel CAZyme-encoding genes that would have promising industrial applications [25]. The use of this combinatorial approach on selected microbial pools from hydrothermal vents extreme habitats has led to the discovery of three archaeal plant-degrading enzymes.

The first predicted GH labeled as EBI-244 is composed of unique mutidomain cellulase in which one domain is similar to GH-5 [115]. The enzyme was cloned and expressed in *E. coli* and shows impressive biochemical features: it works optimally at 109 °C and has a half-life of 5 h at 100 °C, and exhibits high resistance against salts and detergents and was found to be highly active on crystalline cellulose (Avicel) [25].

Another multi-domain glycosidase was discovered in the hot vent of the Kuril archipelago after successful isolation and genome sequencing reassembly of *Thermococcus* sp. strain 2319x1. The enzyme shows an interesting activity in alkaline conditions at pH 8.5. It is made up of three GH domains, one belongs to the GH-5 family and two belong to GH-12, and it has the ability to use diverse substrates such as Avicel, carboxymethyl cellulose, β-1,3 and β-1,4 linked glucose polysaccharides, and mannose- and xylose- based carbohydrates [25,116].

A third example of archaeal enzyme was discovered from microbial communities of Vulcano Island’s extremely shallow marine vents that are mainly composed of more than 96% of archaeal domains in which the hyperthermophilic genera of *Thermococcus* and *Palaeococcus* are highly abundant [117]. The metagenomic analysis revealed the presence of a putative endoglucanase Vul_Cel5A that was expressed and recombinantly produced in *E. coli* and exhibits a remarkable thermostability: it is mostly active at 115 °C under acidic conditions, has a half-life of 43 min at 100 °C and has increased resistance to detergents [25]. Other genes encoding potential biomass-degrading enzymes have been also discovered in partially reassembled genomes. The ability to clone those genes and produce their corresponding enzymes led to the discovery of the archaeal glucosidase Vul_Bgl1A, which demonstrated the greatest activity at 105 °C towards cellobiose and 4-nitrophenyl-D-glucopyranoside [118]. Interestingly, when Vul_Cel5A and Vul_Bgl1A were applied simultaneously, a significant increase in glucose production was observed, indicating that the two enzymes work synergistically [25].

All these detected enzymes would be highly significant candidates for commercial processes, as all of them showed a wide range of biomass substrates and relatively high activity in acidic or alkaline environments, in addition to their strong thermo-activity [25].

Another study that used sequence- and function-based screening methods has been performed to examine thermostable CAZymes, notably cellulases, from an oil reservoir metagenome [119]. The findings of this work have led to the identification of novel thermostable archaeal cellulases that can withstand temperatures of up to 80 °C. Interestingly, the archaeal cellulase F1 was found to have arisen from the fusion of two archaeal cellulases from different families, which could explain its high thermal stability and enhanced activity compared to commercially available enzymes. Besides this high thermostability, its high activity on microcrystalline cellulose for the production of cellobiose and, to a lesser extent, glucose, point to its potentials as an industrial cellulolytic enzyme [119].

Another study has been performed on samples collected from the Tattapani geothermal water of Chhattisgarh, India and used the culture-independent metagenome sequencing approach to investigate the microbial diversity in these hot water reservoirs with temperatures that range from 55 to 98 °C. The metagenomic analysis revealed the presence of archaeal domain in this site in a percentage of 1.1 to 4.8% and detected numerous novel thermostable hydrolytic enzymes, notably β-glucosidase, and xylanase that could have significant importance in the hydrolysis of lignocellulosic plant biomass [120].

## 3. Glycosyltransferases (GTs)

### 3.1. General Features and Reaction Mechanism

Glycosyltransferases constitute a large family of CAZymes that catalyze the transfer of activated forms of monosaccharides to an appropriate acceptor including lipids, proteins, heterocyclic compounds, or other carbohydrate residues to create a diverse range of valuable glycoconjugates, oligosaccharides and polysaccharides [7,121,122]. They are found in both Prokaryotes and Eukaryotes and have been linked to several biological processes in humans, such as cell-signaling and adhesion, cancer, and cell wall biosynthesis [123].

Glycosylation enzymes are classified into two categories based on the glycosyl donor, allowing them to be used in a variety of industrial glycosylation reactions. Leloir enzymes that use sugar mono- or diphosphonucleotide donors, and non-Leloir enzymes that use polyprenol phosphates, polyprenol pyrophosphates, sugar-1-pyrophosphates, or sugar-1-phosphates as non-nucleotide donors [123].

**Figure 6:**
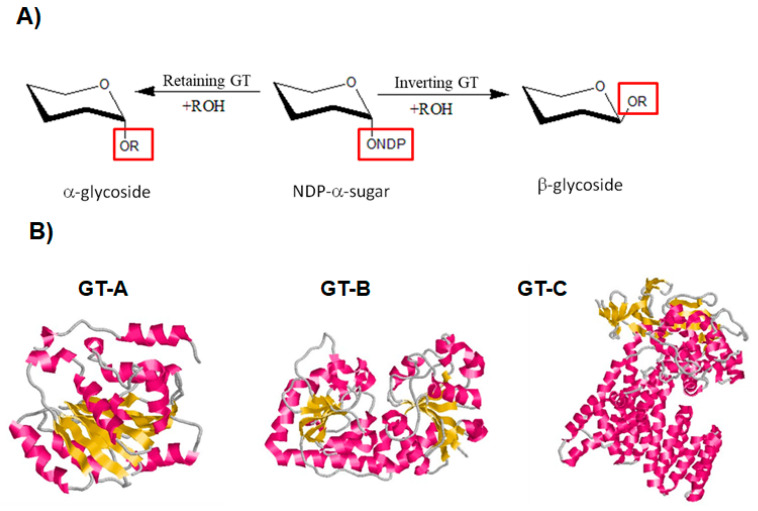
Classification of GTs based on their mechanism for the catalysis of glycosyl group transfer and on their 3D structure [126,127,128]. (**A**) Schematic diagram showing inverting and retaining GTs. (**B**) Classification of GTs based on their 3D structure. Diagram of GT-A fold protein represented by SpsA protein from *Bacillus subtilis* (Protein Data Bank (PDB ID: 1QGQ), diagram of GT-B fold represented by the β-glucosyltransferase of bacteriophage T4, (PDB ID: 1JG7) and diagram of GT-C fold represented by the oligosaccharyltransferase protein from *Campylobacter lari* in complex with peptide substrate and magnesium (PDB ID: 3RCE). The helices are shown in pink, β-strands in yellow and loops in grey.

As for GHs, GTs display two main catalytic mechanisms, either via retaining or inverting the configuration of the anomeric center (Figure 6A) [124,125]. A conserved DXD motif, which is implicated in the binding to a divalent cation, is typically found in both mechanisms of GTs [124].

The majority of GTs adopt one of three folds known as GT-A, GT-B, or GT-C [30]. The GT-A and B folds are made up of two Rossmann-like domains (β/α/β) that bind to the substrate’s nucleotide moiety. Proteins with the GT-C fold are intrinsic proteins that have a variety of transmembrane helix numbers (Figure 6B) [30,124].

According to CAZy database, GTs are classified into 114 families based on the similarity of their amino acid sequences (last accessed July 2021). Unlike GHs, GTs are mainly membrane-bound proteins, are difficult to work with and to purify in sufficient quantities to investigate their biochemical features. However, the cloning and expression approaches have led to a library of recombinant glycosyltransferase that have been essential for their identification and characterization [129].

### 3.2. Hyperthermophilic GTs in Archaea: Biochemical Features and Biotechnological Applications

**Figure 7:**
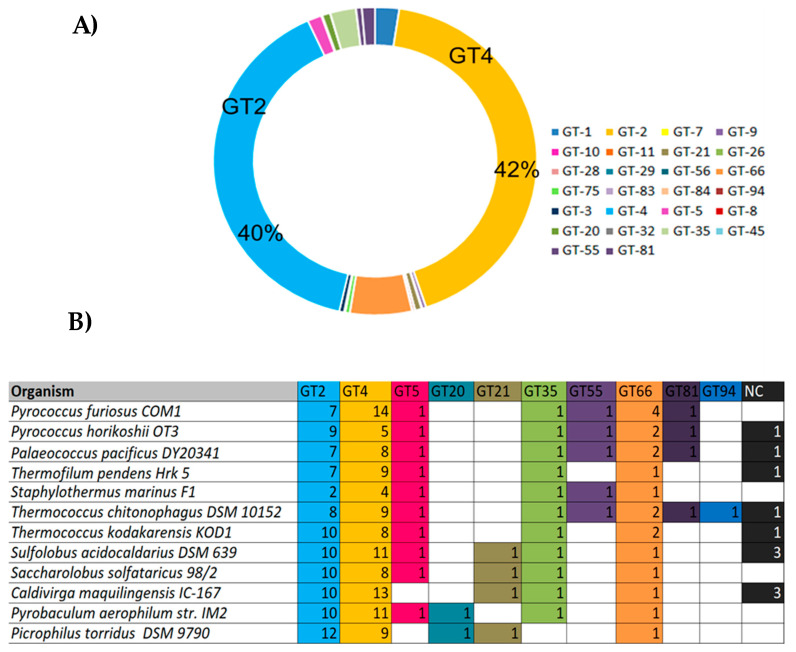
Classification of most abundant archaeal GTs [20]. (**A**) Diagram showing the abundancy of GTs families in Archaea. The sequences from Archaea genomes corresponding to each GT family were retrieved from the CAZY database and the number of occurring sequences for each GT family was determined accordingly; (**B**) Occurrence of GTs in hyperthermophilic Archaea. NC: Non-classified.

Despite the increasing availability of archaeal whole genome sequences, only a limited number of GTs are annotated. They mainly belong to the families GT1, 2, 3, 4, 5, 21, 35, 55, 66, 75 and 81, where GT-2 and GT-4 families predominate and account for more than 40% of all archaeal GTs sequences each (Figure 7A). Few GT-2 and GT-4 families have been characterized and according to the CAZy database, enzymes belonging to these families are commonly involved in cell wall biogenesis, *N*-glycosylation, dolichol phosphate transfer, chitin, cellulose, and sucrose biosynthesis. In addition to the GT2 and GT4 families, the inverting GT66 consisting of a GT using lipid-diphospho-oligosaccharide as a donor is also abundant in archaeal genomes. The GT5 and GT35 from the retaining GTs are also widespread in Archaea genomes and consist of enzymes with glycogen and starch phosphorylase activities. The GT5 members are specific to some thermophilic methanogens such as the Methanococcales, and to hyperthermophilic Archaea such as the Sulfolobales and Thermococcales (Figure 7B). Different enzymatic activities were described within this category such as UDP-Glc: glycogen glucosyltransferase (EC 2.4.1.11), NDP-Glc: starch glucosyltransferase (EC 2.4.1.242), ADP-Glc: starch glucosyltransferase (EC 2.4.1.21), UDP-Glc: α-1,4-glucan synthase (EC 2.4.1.-) and UDP-Glc: α-1,3-glucan synthase (EC 2.4.1.183) [20]. The GT enzymes that have been biochemically characterized are reviewed below and can be divided into two categories: membrane-associated enzymes and soluble enzymes.

### 3.3. Membrane-Associated Archaeal GTs: The Protein N-Glycosylation Pathway

**Figure 8:**
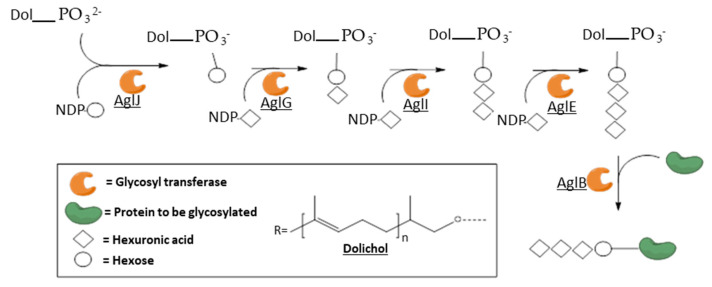
Simplified model of the N-glycosylation pathway in *Haloferax volcanii*, based on Jarrell et al. Some hexuronic residues are methylated and further elongations can occur after the transfer to the protein. AglJ is a dolichol phosphate hexose synthase; AglG, I and E are hexuronyl transferases; and AglB is an oligosaccharyl transferase [132].

The membrane-associated archaeal GTs that have been characterized so far are all part of the complex machinery of protein *N*-glycosylation and are generally annotated as “Agl” for “archaeal glycosylation”. These asparagine-linked glycosylations are among the most common posttranslational protein modifications in all kingdoms of life [130,131]. The whole pathway was deciphered in a few Archaea and shows common features: the first sugar moiety is transferred to a dolichol-(pyro)phosphate carrier by a dolichol phosphate sugar synthase, then iterative additions of sugar moiety are performed by specific GTs using NDP-sugars as glycosyl donors, increasing the length of the oligosaccharide, and finally the oligosaccharidyl chain is transferred to the Asn of the protein to be glycosylated by an oligosaccharyl transferase. Further elongations of the glycan can also exist after the transfer to the protein. The most complete model is the one of *Haloferax volcanii,* summarized in Figure 8 [132].

The stability of archaeal protein combined with the diversity of *N*-linked glycan would have important implications in biotechnology since that this connection may lead to the formation of glycoproteins with special features that can work optimally under specific physically challenging environmental conditions, giving them biotechnological significance in the stabilization of thermoarchaeal protein and in the protection of proteins exposed to highly acidic conditions.

Genes encoding the transferases AglG, I and E and their analogs were identified in several archaeal species, but due to their strong specificity toward complex substrates, i.e., dolichol (pyro)phosphate mono or oligosaccharides, and the difficulty to isolate active enzymes in vitro, the only biochemical features available are mostly related to their substrate specificities [133,134,135,136].

Oligosaccharyltransferases (OST), also referred to as AglB (archaeal Glycosylation protein B), were annotated in all the sequenced genomes of hyperthermophilic archaeal species except *Aeropyrum pernix* and *Methanopyrus kandleri* [137,138,139]. These enzymes, belonging to the GT-66 family, are also extremely difficult to characterize: they have generally between 10 and 16 transmembrane domains and hence are difficult to isolate in vitro, and have strong specificities in terms of nature of the transferred oligosaccharide. Therefore, indirect characterization methods are undertaken such as gene disruption and/or complementation, and glycosylation pattern analysis, for instance, to obtain biochemical information that is generally related only to the nature of their substrates [130,140].

On the other hand, a few dolichol phosphate mannose synthase (DPMS) from Archaea, catalyzing the very first step of these glycosylation pathways, were more deeply biochemically characterized. The first example is the DPMS from *Thermoplasma acidophilum*. The native enzyme was purified after solubilization with various detergents. It catalyzes mannose transfer from GDP-mannose to endogenous and exogenous lipids. From the tested acceptor substrates, it appeared that dolichol phosphate was a significantly better substrate than the others, confirming that the natural activity was DPMS activity. This enzyme displays particular features: it has an almost absolute dependence to divalent cations, the best activity being obtained with Mg^2+^, is mostly active at 65 °C and pH 6.2, and shows a remarkable increase in the activity (three-fold) in the presence of glycerol (1.67 g/L) [141].

Another example of DPMS is the enzyme from *Pyrococcus horikoshii*. This membrane-bound protein from the GT-2 family was cloned and overexpressed in *Saccharomyces cerevisiae*. Like the first one, this enzyme is dependent on divalent cations (Mg^2+^). Its optimal temperature is 60 °C, but it prefers more alkaline conditions than the first enzyme, with a pH of 8.5 [142,143].

After all, by the fact that the archaeal *N*-glycosylation machinery displays high similarity with the eukaryotic one, it can be used as a model system for further research in the field of cell biology, and thus in biomedicine and the pharmaceutical industry.

### 3.4. Soluble GTs

**Figure 9:**
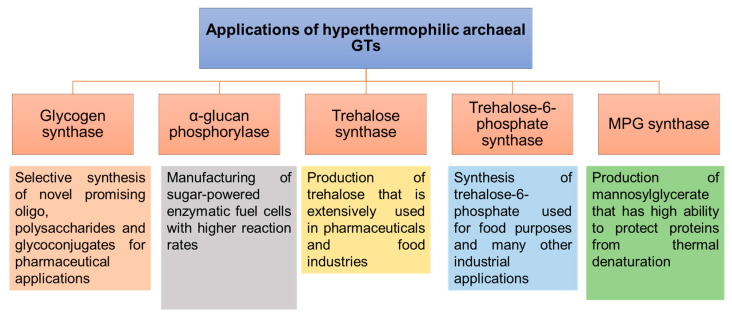
Applications of hyperthermophilic archaeal GTs involved in the synthesis of carbohydrates and glycoconjugates that correspond to glycogen synthases, α-glucan phosphorylases, trehalose synthases, trehalose-6-phosphate synthases and MPG synthases [146,147,148,149,150].

The ability to perform the enzymatic reactions at higher temperatures using thermostable GTs would be advantageous for accelerating reaction rates, increasing the solubility of sugar substrates and possibly for downstream processing and recovery of volatile products [144]. In particular, the remarkable stability of hyperthermophilic archaeal GTs would provide promising applications in the synthesis of carbohydrates and glycoconjugates (Figure 9). Several different activities have been described for the soluble GTs that have been identified and characterized from hyperthermophilic Archaea. Some are involved in energy storage and remobilization (glycogen synthase and α-glucan phosphorylase), while others are involved in the production of protective compounds under stress conditions (trehalose/trehalose-6-phosphate synthases, mannosyl-3-phosphoglycerate (MPG) synthase [145]).

#### 3.4.1. Glycogen Synthase (GS)

Glycogen synthases are retaining glycosyltransferases of the GT-3 and GT-5 families, which polymerize glycogen by catalyzing the transfer of the glucosyl residues from NDP-glucose to the non-reducing end of the growing α-1,4-glucan chain [151]. The thermostability of glycogen synthases isolated particularly from hyperthermophilic Archaea, as well as their ability to catalyze polymerization reactions at high temperatures with higher reaction rates, make them a subject of intense study and offer them a special interest in biotechnology for the selective synthesis of a diverse range of novel natural and synthetic oligosaccharides and polysaccharides with valuable properties for health, food and therapeutic industries, but whose traditional chemical synthesis requires a large number of steps [146].

Two native glycogen synthases from Archaea were isolated and biochemically characterized: one from *Sulfolobus acidocaldarius*, and the other from *Thermococcus hydrothermalis* [152]. The first enzyme belongs to the GT-5 family and displays optimal activity between 70 and 80 °C at pH 7.5. The second is optimally active at 80 °C as well but in a slightly more acidic optimal pH of 5.5. Like the enzyme from *T. hydrothermalis,* this glycogen synthase is able to use either UDP-glucose or ADP-glucose as a glucosyl donor. However, in the presence of ADP-glucose, the enzyme is ten-fold more efficient and shows extreme thermal stability, with a half-life of 2 h at 80 °C and even 6 min at 110 °C.

Later on, a glycogen synthase from *Pyrococcus furiosus* was cloned and heterologously expressed in *E. coli.* This enzyme displays biochemical features close to the enzyme from *T. hydrothermalis*: such as an ideal activity at 80 °C and pH 5. Unlike the two former enzymes, this one has a wide tolerance toward the nature of the nucleotide part of the donor substrate: its activity is very similar with UDP-, ADP-, dTDP- and GDP-glucose [153,154].

#### 3.4.2. α-Glucan Phosphorylase/Maltodextrin Phosphorylases

The α-glucan phosphorylases (or maltodextrin phosphorylase when they have substrate preferences toward oligosaccharides) of the GT-35 family are important enzymes of carbohydrate metabolism. They are involved in the mobilization of stored carbohydrate by catalyzing the reversible cleavage of α-1,4-linkage between pairs of glucose residues at the end of glucose polymers, releasing α-d-glucose-1-phosphate (G-1-P) [155].

Because of their high operating temperatures, thermostable α-glucan phosphorylases are potentially able to produce a diversity of carbohydrates and derivatives and provide significant advantages and greatly facilitated bioprocess design. They also seem to tolerate more variance in substrate structures used for synthesis [147]. They can yield high G-1-P product, which constitutes an important building block for the chemical synthesis of trehalose and glucuronic acid, and amylose derivatives, which have various industrial applications in food and beverage manufacturing [156]. Furthermore, one of the most evolved applications of thermostable α-glucan phosphorylases is their ability to generate bioelectricity from the energy stored in carbohydrate biobatteries that usually work by using low-cost biocatalyst enzymes to directly transform chemical energy from a range of fuels into electricity [147,157]. They can be used in multiple enzymatic conversion processes to catalyze the manufacture of sugar-powered enzymatic fuel cells with faster reaction rates, thereby extending battery life. These biodegradable, high-energy-density, and highly safe batteries would be the next generation of biobatteries, which are highly desirable and in high demand in order to meet the rapidly growing needs of portable electronic devices [157]. 

Native enzymes were purified and characterized from *Thermococcus litoralis* and *Archeoglubus fulgidus* [75,158]. Both enzymes have a very high optimal temperature at 80 °C and 98 °C respectively, work optimally at near-neutral pH at 7.0 and 6.8, respectively. They use pyridoxal-5′-phosphate as cofactor and react on oligosaccharides with a polymerization degree of 4 or more. The two enzymes also display a strong thermostability; for instance, the enzyme from *A. fulgidus* retains all its activity at 80 °C after 120 min, and even at 95 °C with a half-life over 350 min. This later shows a wide pH tolerance, with its activity reaching more than 50% of its maximum in a pH range from 5 to 9.

Two other enzymes from *Pyrococcus furiosus* and *Saccharolobus solfataricus* were cloned and heterologously produced [159]. The first one was expressed in *E. coli* and displays very similar features relative to the native enzymes. Its optimal temperature is at 80 °C, optimal pH is between 6.5 and 7, and total thermostability is at 80 °C over 24 h. The only notable difference is the use of maltotriose as substrate in addition to higher oligosaccharides. The thermostability of this archaeal enzyme, as well as its ability to operate at broad temperature ranges, suggest that it could be useful in biotechnological applications for the degradation of α-glucose polymers [155]. The α-glucan phosphorylases from the hyperthermophilic Archaeon *T. kodakaraensis*, in particular, have demonstrated their unique pyridoxal 5′-phosphate-dependent activity [159].

#### 3.4.3. Trehalose Synthases

Trehalose synthases are retaining GTs that catalyze the formation of trehalose, by transferring a glucose from NDP-glucose to another glucose residue. The resulting product is a non-reducing disaccharide that can be used either as a food additive because it has lower sweetness than sucrose, a moderate glycemic index and low cariogenicity, or can serve as a stabilizer in cosmetics and pharmaceuticals due to its high heat and pH resistance, high glass transition temperature, high water retention capacity and protective effect on proteins [160,161,162].

Several archaeal trehalose synthases from the GT-4 family were heterologously expressed and characterized in *E. coli*. The enzyme from *Pyrococcus horikoshii* has an optimal pH at 5.5 and remains stable between pH 5 and 8. This enzyme, however, is not thermostable: its optimal temperature is under 40 °C, and after 1 h at 55 °C, it retains only 25% of its initial activity. Without acceptor substrate, i.e., glucose, this enzyme displays a slow hydrolysis activity, either on UDP-glucose, *p*-nitrophenyl-α-d-glucopyranoside (pNPGlc), trehalose or glucose-1-phosphate [162].

Another trehalose synthase was described in *Thermococcus litoralis,* but is highly thermostable, and is optimally active at 90 °C in pH 6.5. This enzyme is Mg^2+^ dependent and can catalyze the reaction in a reversible manner. It can also use different NDP-glucose donor substrates: the best activity is obtained with ADP-glucose, but it can also use UDP- or GDP-glucose with lower efficiency [163].

Finally, a thermostable, Mg^2+^ dependent, trehalose synthase was described in *Thermoproteus tenax*. This enzyme, however, can only catalyze trehalose synthesis and not the reverse reaction. It functions optimally at 80 °C and can also use UDP- and ADP-glucose as donors, but with a preference for UDP-glucose in this case (10% of the activity with ADP-glucose) [164].

##### Trehalose 6-Phosphate Synthases

Trehalose-6-phosphate is one of the most important precursors in trehalose biosynthesis, as it is formed by the enzyme trehalose-6-phosphate synthase and then dephosphorylated to trehalose by trehalose-6-phosphate phosphatase [165].

This activity has been extensively described in Eukaryotes and Bacteria, but the only characterized trehalose-6-phosphate synthase from Archaea is from *Thermoplasma acidophilum* [148]. This enzyme was cloned and heterologously expressed in *E. coli* and is mostly active at 60 °C and pH 6.0. The enzyme displays remarkable stability: it has a half-life of 6 h at 60 °C and retains half of its initial activity when incubated for 6 h at pH of either 2 or 9, and even treatment with denaturing agents has little effects where 70 to 80% of the initial activity remains with 10% of methanol, ethanol, isopropanol, mercaptoethanol or sodium dodecyl sulfate, or with a 10 mM EDTA, urea or dithiothreitol. The activity of this enzyme is increased in the presence of divalent cations (Mg^2+^, Zn^2+^, Co^2+^) but these cofactors are not mandatory. In addition to its stability, this enzyme displays an interesting substrate tolerance: it can accept UDP-glucose as well as ADP- and GDP-glucose as donor substrates, with a relative activity of 50% and 20% of the maximal activity respectively. In addition to glucose-6-phosphate, the enzyme can also transfer a glucose moiety to mannose-6-phosphate with a relative activity of 40%, fructose-6-phosphate or glucosamine-6-phosphate with 10% of relative activity for these two latter acceptors [148].

This archaeal trehalose-6-phosphate enzyme could represent a potential candidate in biotechnological applications for the synthesis of trehalose-6-phosphate that regulates starch metabolism in plants or for the transgenic improvement of tolerance of abiotic stress in organisms [165].

#### 3.4.4. Mannosyl-3-Phosphoglycerate (MPG) Synthases

MPG synthases are retaining GT-55 that catalyze the formation of α-mannosyl-3-phosphoglycerate, by transferring a mannose residue from GDP-mannose to 3-phosphoglycerate. The resulting mannosylglycerate product is synthesized in vivo as a protein thermoprotectant, allowing proteins to be protected from heat damage and giving it the potential to be used as a stabilizer of biomaterials to protect enzymes against stress as well as in various biotechnological and pharmaceutical applications [166].

Two enzymes from the hyperthermophilic Archaea *Pyrococcus horikoshii* and *Paleococcus ferrophilus* were cloned and expressed in *E. coli* [150,167]. They belong to the GT-55 family and display similar properties. They have an optimal pH between 6.4 and 7.4, and an optimal temperature over 90 °C, at 98 °C and 90 °C, respectively, with a very strict substrate specificity. They show also high thermal stability, with half-lives of 16 and 18 min at 98 °C and 83 °C, respectively. Additional features have been investigated with *P. ferrophilus* enzyme: it is Mg^2+^ dependent and is inhibited by NaCl and KCl [167]. Further efforts can be investigated in the future to convert the recombinant *E. coli* into an efficient cell factory to produce MG at low cost for large-scale applications.

## 4. Conclusions

The vast majority of CAZymes from hyperthermophilic Archaea in under-explored habitats have yet to be discovered and biochemically characterized. The discovery and increasing number of novel, highly thermostable CAZymes from hyperthermophilic Archaea will open up new possibilities for industrial processes as they present an innovative solution for those that are carried out at high temperatures. While many glycosyl hydrolases have been extensively characterized and their potential role in biotechnology are being investigated, the genomes of many hyperthermophilic Archaea encode valuable glycosyl transferases that are of great potential to the biotechnological industry. These enzymes can offer numerous commercial applications in the synthesis of glycoconjugates and the chemoenzymatic and recombinant biosynthesis of novel sugar biomolecules. Further biochemical and structural exploration would link functional activity to genomic predictions and expand our known repertoire for hyperthermophilic CAZymes.

## Data Availability

Not applicable.
